# Richmond Academy of Medicine and Surgery

**Published:** 1896-10

**Authors:** Mark W. Peyser

**Affiliations:** Secretary and Reporter


### Society Reports.

### RICHMOND ACADEMY OF MEDICINE AND SURGERY.

Regular Meeting, August 25, 1896, Hall of Y. M. C. A.

Dr. Landon B. Edwards, President, in the Chair. Dr. Mark W. Peyser, Secretary and Reporter.

Dr. J. Allison Hodges read a paper on

"THE NOT USUAL EFFECTS OF THE BROMIDES."

In the discussion which followed, Dr. J. N. Upshur remarked that Dr. Hodges' paper was one of great value to the Academy and the profession at large. He entirely agreed with the views expressed in it. For many years he has believed that the profession is as much too prone to give the bromides as morphine. He was peculiarly in sympathy with the statement as to the indiscriminate use by the profession and laity of the various proprietary preparations containing these agents. He has been in the habit of prescribing the bromides with more or less frequency for several years, because he did not wish to prescribe them empirically, and because of their effect on the circulation. He hoped the article would be widely read, and that the profession would heed the warning given.

Dr. Wm. S. Gordon agreed with both gentlemen preceding him. Of course, it is routine practice to give the bromides in epilepsy. He narrated the case of an epileptic girl who, after taking considerable doses, developed all the prominent symptoms mentioned by Dr. Hodges; and at the time, he wondered how far they were due to the medicine or the disease. He asked if, under these circumstances, had Dr. Hodges substituted hydrobromic acid, and, if so, the symptoms when pushed to the farthest point? He has used hydrobromic acid with good effect, but he had never continued it long enough to contrast its effects with those of the bromide salts.

The President agreed with most of the points made by Dr. Hodges; and yet he could not allow the enthusiasm occasioned by the valuable paper to overshadow the good results often obtained from the use of the bromides. He did not know how we could manage without them. It was not usual, in his experience, for patients to desire a continuance of their use; he wished to do away with the idea that there is a bromide habit, due to a desire on the part cf the patient to take this agent. Large doses have their value in certain cases, but, ordinarily, these are not called for. As a rule, the bromide treatment is the most valuable for epilepsy. In the experience of those treating *petit mal*, it is of little value, but in the *grand mal* it is of utmost value.

Suicidal and homicidal tendencies are noted, not in the cases where there is deliberation and cunning, but in the careless, somnambulistic, etc. At any rate, he was not prepared to say these tendencies came from bromism. The epileptic is often dangerous to himself and others, even without treatment by bromides.

DR. HODGES, in closing the discussion, agreed with Dr. Edwards in the efficacy of the bromides in some cases, but limited their greatest utility to cerebral disorders from overaction. He differed with him, however, as to the danger of patients forming a "bromo habit," and cited the large and increasing use by the laity of the numerous bromo preparations, as was evidenced by their enormous consumption within the past few years. He doubted the necessity of the bromides or their ultimate benefit to the patient in the treatment of epilepsy, and cited instances to substantiate this assertion, claiming that other drugs, or even the bromides differently administered, would accomplish the same results. He had used hydrobromic acid as a sedative and hypnotic, and had found it useful, but had never pushed it to its physiological effects. It was probable, however, that under such conditions it would prove an irritant to the gastric mucous membrane.

### REPORTS OF CASES.

### CONVULSIONS AND HEMI-HYPERÆSTHESIA PRECEDING TYPHOID FEVER.

DR. UPSHUR reported the case of a woman, age 20. Two months ago, he was sent for at night and found she had had a severe convulsion before his arrival. When seen, her mind was not entirely clear—was very nervous, presenting symptoms of a markedly hysterical condition. There had been much malaise for a week previous. The prominent symptom was hyperæsthesia of the whole right half of the body and head, especially marked in the arm and leg. Simple touch would throw the group of muscles in the neighborhood into violent spasms. Along with this, for a week, there had been a regular rise and fall of temperature—99° in the morning to 101° in the afternoon. At the end of the week, the hyperæsthesia seemed to subside, but she was nervous and sleepless; the tongue was heavily coated, pulse frequent, abdomen distended and tender, with gurgling in the right iliac fossa. In fourteen or fifteen days, symptoms of marked typhoid made their appearance. From that point the case was typical, with a tendency to development of coma vigil. Coma did supervene, and the patient died on the twenty-seventh day. Obstinate constipation persisted throughout the course of the disease. Every action was procured by an enema. The onset of the trouble was the most curious he had ever seen.

DR. UPSHUR also reported the next case, *concerning which he would like to have an expression of opinion.* Woman, age 40; seen ten days ago in consultation. She had been in delicate health for about a year, and there was obscurity as to her trouble. During last spring, she was satisfied as to the existence of pregnancy. Taking some medicine given by a negress, she aborted and recovered. Later, she was seized with violent pain in the right groin, extending around the crest of the ilium down the thigh—being particularly painful in the knee, and paroxysmal in character, especially pronounced in the morning and afternoon. There were rises and falls of temperature at various times of the day, from 99° to 104-5° F.

During an attack, when first seen, he had found an over-distended bladder. The urine was albuminous, but no trouble ensued upon drawing it off.

Ten days ago she was removed to the Old Dominion Hospital, the temperature rising as a result two degrees, falling in a day or two.

The administration of chloroform did not effect a complete relaxation of the abdominal muscles, but a thorough examination of the pelvis revealed no trouble, unless, possibly, a pre-existent endometritis. A point just above Poupart's ligament on the right side, showed evidence of tenderness. *Per vaginam*, he could approximate the fingers so that the thickness of the abdominal wall could be made out. Examination of the urine by Mr. Blair evidenced slight albumin, but was negative otherwise.

[CONTINUED TO NOVEMBER ISSUE.]

